# Methamphetamine Lab Explosion: A Pediatric Emergency Medicine Case

**DOI:** 10.7759/cureus.30968

**Published:** 2022-11-01

**Authors:** Jean Pearce

**Affiliations:** 1 Pediatric Emergency Medicine, Medical College of Wisconsin, Milwaukee, USA; 2 Pediatric Emergency Medicine, Children's Wisconsin, Milwaukee, USA

**Keywords:** decontamination, airway management, inhalational injury, methamphetamine, simulation, pediatric emergency

## Abstract

Children exposed to the manufacture of illegal drugs are at risk for multiple medical problems. Providers need to be able to recognize and manage the complications from these exposures because early intervention can be crucial to decreasing morbidity and mortality. In this simulation case, a 3-year-old patient is brought to the emergency department (ED) after a house fire due to a methamphetamine lab explosion. The goals of this case are to provide the learners with the training and opportunity to manage a toxic chemical exposure by applying principles and methods of decontamination, and to manage an inhalational injury with rapidly progressive airway edema. These events being rare, this simulation gives learners crucial experience with a high-stakes medical condition.

## Introduction

Methamphetamine (meth) is an illegal and highly addictive stimulant. Meth use and manufacture in the USA has remained a problem over the past 10 years. According to the Drug Enforcement Agency (DEA), more than 9,000 meth clandestine laboratory incidents including labs, dumpsites, and chemical seizures occurred in 2014 [1]. Operational illegal labs pose a great risk to the public through both, exposure to toxic chemicals or byproducts, and fire or explosion due to the volatile nature of the chemicals used. Chemicals used can include corrosives/irritants (hydrochloric acid, sodium hydroxide, and anhydrous ammonia), solvents (ether, toluene, denatured alcohol, and Freon), drugs (ephedrine, pseudoephedrine, phenylpropanolamine) and other poisons (mercury, lithium metal, and lead) [2,3]. Patients can exhibit a variety of symptoms following an explosion or fire from a meth lab as a result of exposure to contaminated substances. These can include shortness of breath, cough, chest pain, pulmonary hemorrhage, severe burns, dizziness, headache, disorientation, and nausea [2,3]. Most of these toxic chemicals do not have specific antidotes. Thus, treatment consists of decontamination and supportive care.

The decision to start decontamination is based on a variety of factors including signs and symptoms of exposure, visible evidence of contamination on the patient’s skin or clothing, and proximity of the patient to the exposure. The key elements of decontamination in this situation include the use of PPE for providers, removing and placing patient clothing in sealed bags, and water-based decontamination which should consist of low pressure, high volume, tepid water for a duration of at least 30 seconds but no longer than 3 minutes [4]. Children are at the greatest risk of exposure to toxic chemicals due to their developmental stage which puts them at greater risk given their hand-to-mouth behavior and play on the ground. Additionally, children have a higher surface-to-volume ratio which can lead to increased absorption of toxins through dermal contact compared to adults [5].

Although only 3-5% of all burns in children are life-threatening, a large portion of these are associated with house fires [6]. A small proportion of these life-threatening burns in children involve inhalational injury, though this is more frequent in children compared to adults due to their inability or unwillingness to leave a room during a fire. Mortality for children with inhalational burns is 16% [7]. Inhalational burns occur when children in a fire inhale hot gases that burn the upper airway. This may eventually result in progressive edema and airway obstruction. Children at the highest risk may exhibit hoarseness, stridor, facial burns, singed facial hair, or soot around the nares [6,8]. However, the absence of these signs does not rule out airway burns. Thus, the ED provider must be vigilant and prepared for any changes in patient status. Early intubation in these patients may prevent significant difficulties later with airway management as airway edema progresses.

The goals of this scenario are to provide learners with the training and opportunity to manage a toxic chemical exposure by applying principles and methods of decontamination and to manage an inhalational injury that can have rapidly progressive airway edema requiring critical early intervention in a pediatric patient. As these may be rare events not encountered by all participants during training, this session gives learners crucial experience with a high-stakes medical condition.

ED providers should be familiar with signs and symptoms of a toxic chemical exposure, indications for PPE and strategies to protect medical personnel from secondary exposures, patient decontamination procedures, performing a primary survey for signs and symptoms of an inhalational burn injury, management of airway emergencies including endotracheal intubation, and obtaining the assistance of difficult airway teams. This simulation focuses on these objectives and skills and is appropriate for intermediate to advanced learners familiar with Pediatric Advanced Life Support (PALS) algorithms.

## Technical report

This simulation case is designed to last approximately 1 hour inclusive of the debrief. In order to make this simulation as realistic as possible, it should take place in either an actual ED critical care room or a simulation room that is set up to be identical to one with standard resuscitation equipment available (PPE, oxygen, intubation supplies, medications, vascular access supplies, etc.). This case was designed to be used with a high-fidelity simulation child mannequin but could easily be adapted to use with a low-fidelity mannequin. The scenario was led by a facilitator experienced in pediatric resuscitation and simulation. Team roles included team lead, bedside provider, airway provider, bedside nurse, pharmacist, family liaison, EMS provider, and simulation facilitator.

This scenario has been run with PEM fellows as the primary participants. Pediatric and emergency medicine residents have participated in observer roles. A total of 15 learners participated in this case. Actors played by PEM faculty filled the roles of the EMS provider and the bedside nurse. Physical exam findings were provided verbally by the facilitator when unable to be demonstrated by the mannequin. A critical actions checklist was developed for the case and a post-simulation participant evaluation was given to all learners following the simulation.

Below are the learning objectives for the simulation case:

1- Recognize and manage a toxic chemical exposure

a. Identify risk factors for a toxic chemical exposure by obtaining a targeted history

b. Identify patient and medical personnel signs and symptoms of a toxic chemical exposure

c. Apply appropriate Personal Protective Equipment (PPE)

d. Institute appropriate patient decontamination including removal and bagging of clothing and water-based decontamination

2- Recognize and manage an inhalational burn injury

a. Recognize patient risk factors for inhalational injury

b. Identify patient signs and symptoms of inhalational injury

c. Identify additional resources required (respiratory therapy, pharmacy, and subspecialty consultation for anticipated difficult advanced airway management, etc.)

d. Demonstrate endotracheal intubation

3- Demonstrate effective leadership and communication skills

a. Identify team leader and assign team member roles

b. Summarize periodically to develop a shared mental model among the team

Pre-briefing

Prior to the start of the simulation, participants are oriented with a “pre-briefing.” During the pre-brief, any introductions are made if required. The facilitator should explain that this is a safe environment for learning and establish ground rules for participants such as mutual respect. Participants should be oriented to the overall timeline for the session and logistics of the room including simulator capabilities and available equipment. The facilitator should discuss confidentiality if applicable and clearly outline participant expectations for the simulation including a fiction contract.

Case

**Figure 1:**
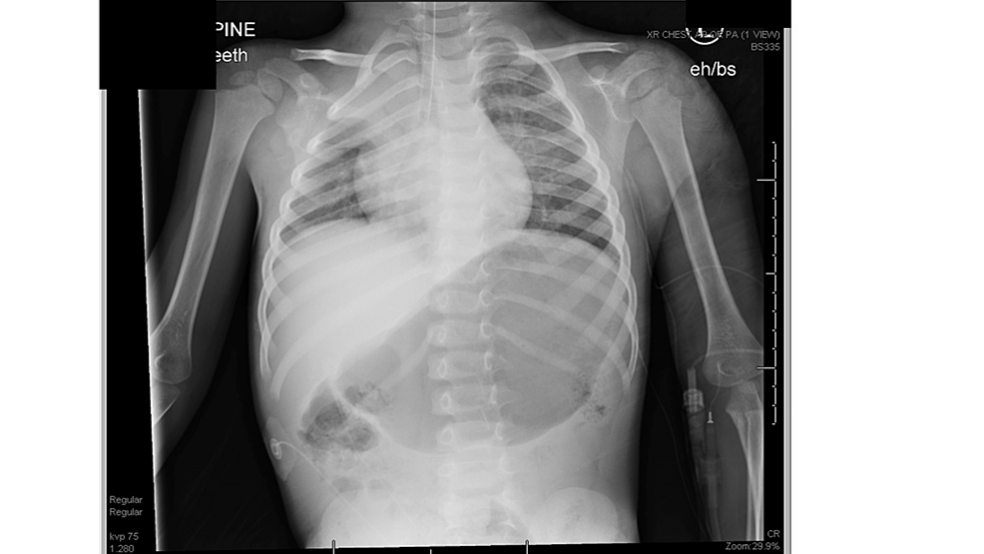
Portable chest X-ray

This simulation case is a 3-year-old male presenting via EMS after a house fire due to a methamphetamine lab explosion. The case begins with the facilitator reading the EMS page (Table 2) and proceeds with the learners having a few minutes to prepare for patient arrival. The mannequin will be transported by EMS into the ED or simulated-ED room where monitors are applied and handoff occurs. When requested, the facilitator provides the history, vitals, and physical exam findings. The case advances based on the participant's actions with the facilitator following the stepwise case progression (Table 3). Labs and imaging may also be provided when requested by the learners (Table 4 and Figure 1). If available, a second faculty observer may fill out the critical actions checklist during the simulation to provide more targeted feedback during the debrief (Table 5).

**Table 1:** Facilitator overview, pediatric emergency medicine simulation: methamphetamine lab explosion. ED = emergency department, PICU = pediatric intensive care unit, EMS = emergency medical services, PPE = personal protective equipment, RSI = rapid sequence intubation

Patient: Andrew, Age: 3 years, Weight: 15 kg	
Brief case description	The patient is a 3-year-old previously healthy male who is brought to the ED by EMS personnel. He was found outside on the lawn of a house fire which was due to a methamphetamine lab explosion. He initially is crying and has a cough with singed clothing that has a strong chemical smell. Upon arrival at the ED, the EMS personnel begin to display symptoms of toxic exposure including nausea, dizziness, cough, and shortness of breath. The learners must recognize the toxic chemical exposure and initiate measures for staff protection and patient and provider decontamination. EMS personnel will not require medical attention from the team. After appropriate decontamination occurs the patient exhibits worsening respiratory symptoms including stridor and hypoxia. The learners must recognize the presence of and manage inhalational injury requiring advanced airway management. The case will conclude after intubation and transfer to the PICU.
Participant roles (required)	Simulation facilitator, team lead, airway provider, bedside provider, bedside nurse, EMS provider
Participant roles (optional)	Co-simulation facilitator, family liaison, pharmacist, simulation technician, critical action checklist reviewer, observers
Supplies	Monitors, oxygen, PPE (gowns, gloves, eye protection, plastic bags for contaminated clothing), bag-valve-mask set-up, suction, intubation supplies, airway adjuncts (supraglottic airway, oropharyngeal airway, etc.), medications, IV supplies
Learning objectives, 1	Recognize and manage toxic chemical exposure. A) Identify risk factors for toxic chemical exposure by obtaining a targeted history. B) Identify patient and medical personnel signs and symptoms of toxic chemical exposure. C) Apply appropriate PPE. D) Institute appropriate patient decontamination including removal and bagging of clothing and water-based decontamination.
Learning objectives, 2	Recognize and manage an inhalational burn injury. A) Recognize patient risk factors for inhalational injury. B) Identify patient signs and symptoms of inhalational injury. C) Identify additional resources required (respiratory therapy, pharmacy, and subspecialty consultation for anticipated difficult advanced airway management, etc.). D) Demonstrate endotracheal intubation.
Learning objectives, 3	Demonstrate effective leadership and communication skills. A) Identify team leader and assign team member roles. B) Summarize periodically to develop a shared mental model among the team.
Critical actions, Clinical state #1: Scenario introduction/EMS page	1. Identify a team leader and assign roles. 2. Identify and obtain any immediate resources needed.
Critical actions, Clinical state #2: Initial patient presentation	1. Perform a primary survey and apply monitors. 2. Apply oxygen. 3. Obtain IV access and administer an isotonic 20 cc/kg fluid bolus. 4. Obtains labs including electrolytes, glucose, blood gas, and carboxyhemoglobin 5. Obtain a targeted history.
Critical actions, Clinical state #3: Toxic chemical exposure/Decontamination	1. Identify a toxic chemical exposure. 2. Apply appropriate PPE and utilize decontamination procedures
Critical actions, Clinical state #4: Inhalational injury/Airway management	1. Identify inhalational injury. 2. Identify appropriate additional resources (respiratory therapist, difficulty airway team). 3. Order appropriate RSI medications. 4. Perform endotracheal intubation.
Critical actions, Clinical state #5: Patient disposition	1. Transfer to PICU and provide verbal handoff to the critical care team.
Ideal Scenario Flow	The learners select a team leader, assign additional team roles, and prepare any supplies needed in advance. Once the patient arrives, the team places the patient on monitors, applies oxygen, performs a primary survey, obtains IV access, draws lab work, orders a fluid bolus, and obtains a focused history from EMS. The EMS provider then develops symptoms of dizziness, nausea, and shortness of breath. The team recognizes a toxic chemical exposure, dons appropriate PPE, and institutes decontamination procedures for the patient and EMS provider including clothing removal and bagging and water decontamination. The EMS provider will not require medical treatment by the team and they will be informed the EMS provider is being cared for by another team. The team notes the patient’s worsening respiratory distress, obtains resources for advanced airway management, and endotracheally intubates the patient. The PICU is called for admission and a concise and accurate summary of the patient and events is provided by the team leader.
Anticipated Management Mistakes	1. Failure to obtain additional history from EMS. 2. Failure to recognize a toxic chemical exposure. 3. Unfamiliarity with decontamination procedures. 4. Failure to recognize the need for intubation.

**Table 2:** Scenario Introduction, State #1 ETA = estimated time of arrival

Scenario Introduction	
EMS Page, State #1	3-year-old boy found outside of a house fire, singed clothes. No IV. HR 135, RR 30, BP 100/70, SpO2 98%. ETA 3 minutes.

**Table 3:** Stepwise progression of care EMS = emergency medical services, ABCs = airway, breathing, circulation, GCS = Glasgow coma score, HR = heart rate, SpO2 = oxygen saturation, BP = blood pressure, RR = respiratory rate, Temp = temperature, POC = point of care, ETT = endotracheal tube, ETCO2 = end tidal CO2

Stepwise Progression of the Case		
Intervention/Time Point	Change in case	Additional Information
State #2, Initial patient presentation. The EMS provider brings the patient to the ED room and provides the handoff, providers wait at the bedside.	Learners should obtain handoff from the EMS provider and ask any additional clarifying questions/obtain a targeted history.	EMS handoff: “Andrew is a 3-year-old-male who was found sitting on the front lawn at a house fire. No interventions were done en route.” If specifically asked, the EMS provider can give the following information: “Police were on the scene first, stated that neighbors called 911 after a loud “boom” was heard and saw smoke. Andrew was inside the house at the start of the fire and was extricated by a neighbor. He had no loss of consciousness. Two unconscious adults were extricated and taken to a neighboring hospital.”
Primary survey/patient evaluation	Learners should perform a primary survey including ABCs, apply monitors, obtain vital signs, and examine the patient.	Vitals: HR 145, SpO2 97%, BP 100/70, RR 30, Temp 37.0 C General appearance: Patient is sitting on the gurney, crying and anxious. He is disheveled and has no shoes on. His clothes are singed, and he has a strong chemical smell. Primary survey: Airway: patent, no soot in the nares. Breathing: tachypneic, clear. Circulation: tachycardic, pulses are +2, the capillary refill is 2-3 seconds. Disability: GCS 14 (V4-irritable and crying, E4-spontaneous eye opening, M6- spontaneous movement)
IV access was obtained, fluids started, and lab results were requested. State #3 is triggered after the isotonic fluid bolus is completed	Learners should request IV access and administer a 20 cc/kg isotonic fluid bolus. Lab results are available upon request, if not requested can have the bedside nurse ask when placing the IV: “Do you want me to collect any labs?”	POC labs were immediately available: pH 7.34, pCO2 45 mmHg, pO2 100 mmHg, Na 135 mmol/L, K 3.5 mmol/L, hematocrit 40%, glucose 115 mg/dL. See Table 4 for additional labs available at a later time.
State #3, Decontamination. The EMS provider should state he/she is dizzy, nauseous, and short of breath, and sit down in a chair in the room. Physical exam and vital signs are the same as State #2. State #4 is triggered after decontamination procedures are completed.	Learners should recognize that there is toxic chemical exposure affecting the EMS provider, apply appropriate PPE (gloves, eye protection, mask, gown) and institute decontamination procedures including removal of and bagging the patient’s clothing. The team does not need to demonstrate full decontamination procedures on the mannequin but should remove clothing and verbalize additional steps including the need to decontaminate the EMS provider.	If the team ignores the EMS provider’s symptoms, the bedside nurse can state that he/she is also experiencing symptoms.
State #4, Airway management. Repeat vital signs are HR 150, RR 35, SpO2 91%, BP 100/70. Physical exam was significant for mild stridor, mild accessory muscle use, and increasing anxiety.	Learners should recognize evolving airway edema from an inhalational injury with hypoxia, tachypnea, increased work of breathing, stridor, and increased patient anxiety. Learners should call for additional resources (respiratory therapy, difficult airway team). If the team does not proceed with intubation, facilitator should cause the patient to have progressive hypoxia, stridor, and respiratory distress.	ETT: 4.0-4.5 cuffed. Blade: Miller or Macintosh #2. Intubation medications for sedation: etomidate 0.3 mg/kg, ketamine 1-2 mg/kg, propofol 1-1.5 mg/kg. Intubation medications for paralysis: rocuronium 1-1.2 mg/kg. If a chest X-ray is requested post-intubation, the ETT is located just above the carina. See Figure 1. ETCO2: 35.
State #5, Patient disposition. Sign-out and transition of care to the PICU/25 minutes. Vitals: HR 120, SpO2 100%, BP 100/70, RR 20, Temp 37.0 C. Physical exam: endotracheal tube in place, breath sounds clear bilaterally, regular rate and rhythm, pulses +2, capillary refill 2-3 seconds, intubated and sedated, GCS 3.	Learners should determine disposition of this patient to the PICU and provide appropriate verbal sign-out to this team.	The facilitator can play the role of the PICU physician. Sample sign-out: “3-year-old male presenting after a housefire from likely methamphetamine lab explosion requiring external decontamination and with progressive signs of airway edema including stridor, hypoxia and respiratory distress now intubated and sedated.”

**Table 4:** Labs POC = point of care, pCO2 = venous carbon dioxide; pO2 = venous oxygen; Na = sodium; K = potassium; Cl = chloride; Glu = glucose; Lac = lactate; Creat = creatinine; Hct = hematocrit; WBC = white blood cells; Hbg = hemoglobin; Plt = platelets; CO2 = bicarbonate; BUN = blood urea nitrogen; Mg = magnesium; Phos = phosphorous; Total Bili = total bilirubin; AST = aspartate aminotransferase; ALT = alanine transaminase

POC labs immediately available	Sent to lab
pH: 7.35	WBC: 8.0 K/µL
pCO2: 45 mmHg	Hgb: 13 g/dL
pO2: 100 mmHg	Plt: 350 THOU/µL
Na: 135 mmol/L	
K: 3.5 mmol/L	Na: 134 mmol/L
Glu: 115 mg/dL	K: 3.9 mmol/L
Hct: 40%	Cl: 105 mmol/L
	CO2: 22 mmol/L
	BUN: 6 mg/dL
	Creat: 0.5 mg/dL
	Gluc: 112 mg/dL
	Albumin: 4.5 g/dL
	Mg: 3.0 mg/dL
	Phos: 4.0 mg/dL
	Total bili: 1.0 mg/gL
	AST: 35 U/L
	ALT: 40 U/L
	Carboxyhemoglobin: 4%

**Table 5:** Critical Actions Checklist

Action	Completed?		
	Yes	Partially	No
Assign team leader and team member roles			
Perform a primary survey (airway, breathing, circulation, disability, exposure)			
Elicit a targeted history			
Identify and manage a toxic chemical exposure			
Identify inhalational injury and perform endotracheal intubation			
Determine a patient disposition and summarize the events for the admitting provider			

Debriefing

After the simulation, the learners participated in a 30-35 minute debrief led by the facilitator. If possible, it is ideal to have the debrief occur in a room separate from the simulation itself. The facilitator leading the debrief was trained in simulation and debriefing techniques and utilized the PEARLS (Promoting Excellence and Reflective Learning in Simulation) method [9]. The debrief was structured into four phases: Reactions, Description, Analysis, and Summary. Sample questions/topics specific to this case that facilitators can use during each phase to direct the debrief were developed into a debriefing guide (Table 6).

**Table 6:** Debriefing guide PEARLS = Promoting Excellence and Reflective Learning in Simulation

Debriefing Guide	
Stage	Discussion Prompts
Reactions	“How did that feel?” “How do you feel?” “What are your overall reactions?”
Description	“Can someone summarize the events of the simulation for us?” “What are the main events of this case?” “What was going on with the patient in this case?”
Analysis	With the PEARLS method, instructors may select a variety of different debriefing methods ranging from learner self-assessment to focused facilitation to facilitator-provided information based on the time, rationale and content being addressed. While the topics covered should directly tie back to the learning objectives of the case, if additional questions or concerns not addressed in the objectives are raised by the learners these can be explored too depending on time constraints.
Summary	“I’d like to end the debrief session by having everyone tell me a take-away they learned from today’s session.” “What are some key learning points you learned today?” “What are you going to do differently in the future because of today’s session?”

Post-scenario Didactics

Following the scenario and the debrief, the learners were provided with an electronic didactic presentation with emphasis on reinforcing learning objectives and key concepts related to medical management. Figures are provided below with explanations addressing the objectives.

Objective 1: Recognize and manage a toxic chemical exposure

A: Identify risk factors for toxic chemical exposure by obtaining a targeted history

B: Identify patient and medical personnel signs and symptoms of a toxic chemical exposure

C: Apply appropriate Personal Protective Equipment (PPE)

D: Institute appropriate patient decontamination including removal and bagging of clothing and water-based decontamination

**Figure 2:**
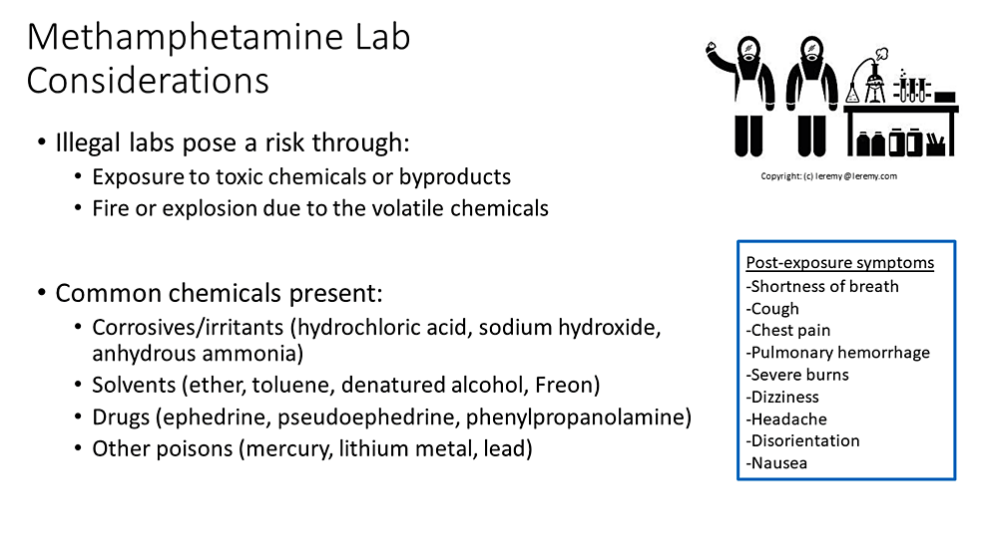
Methamphetamine lab considerations slide

**Figure 3:**
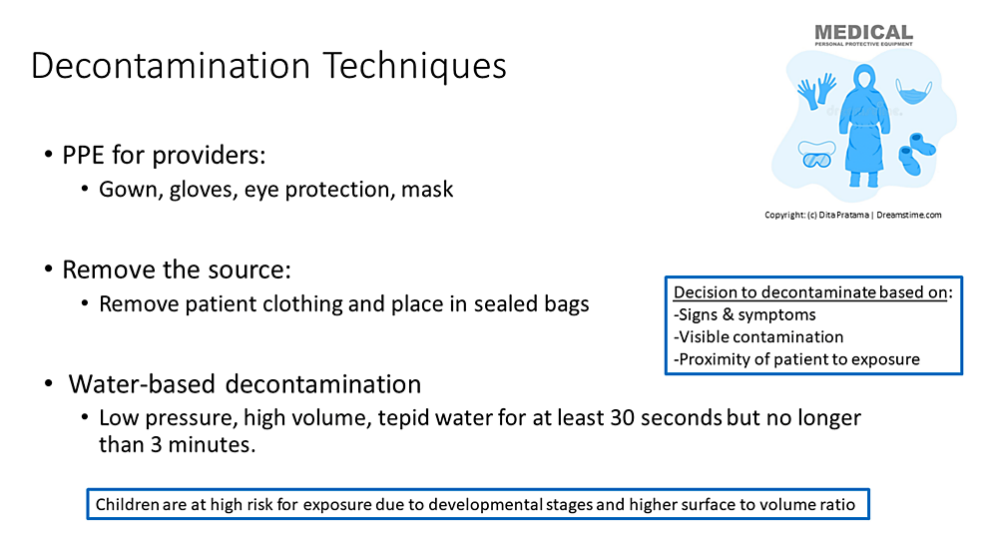
Decontamination techniques slide

Exposure to methamphetamine labs can pose a risk to children via exposure to toxic chemicals and byproducts and fire or explosion due to the volatile nature of these chemicals. There are many different ways people may use to manufacture methamphetamines and so the potential chemical exposures can be diverse including corrosives or irritants (hydrochloric acid, sodium hydroxide, anhydrous ammonia), solvents (ether, toluene, denatured alcohol, Freon), drugs (ephedrine, pseudoephedrine, phenylpropanolamine), and other poisons (mercury, lithium metal, lead) [2,3]. Because providers caring for these exposed children will often not have specific information about the exact substances, it is best to keep a broad approach, though the majority of these substances do not have antidotes, and exposure is managed through symptomatic and supportive care. Post-exposure symptoms to watch for in exposed children include shortness of breath, cough, chest pain, pulmonary hemorrhage, burns, dizziness, headache, disorientation, and nausea. Children may be at increased risk for exposure due to their developmental stages and hand-to-mouth behaviors and their higher surface-to-volume ratio increasing their absorption of toxins [10]. Decontamination is an important initial step to remove any offending agents before treatment can begin. Decontamination techniques should include consideration of healthcare personnel if they have been exposed to clothing or objects/toys the child has from the environment (Figures 2, 3).

Objective 2: Recognize and manage an inhalational burn injury

A: Recognize patient risk factors for inhalational injury

B: Identify patient signs and symptoms of inhalational injury

C: Identify additional resources required (respiratory therapy, pharmacy, and subspecialty consultation for anticipated difficult advanced airway management, etc.)

D: Demonstrate endotracheal intubation

**Figure 4:**
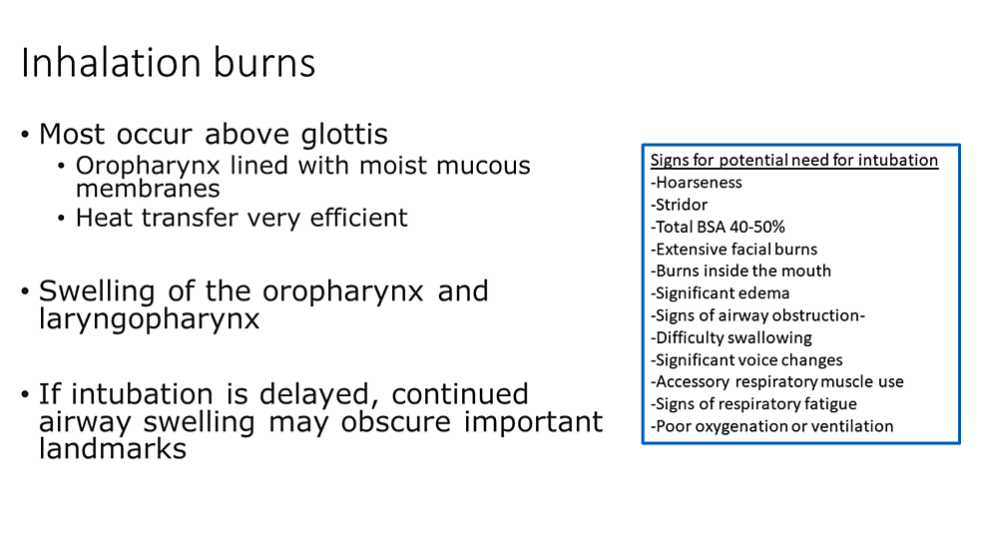
Inhalation burns slide

Although rare, inhalational burns can become a life-threatening emergency in children. Most life-threatening burns in children are associated with house fires [6]. When children inhale hot gases, they can sustain burns to the upper airway which is lined with moist mucous membranes resulting in very efficient heat transfer. Most of these burns occur above the glottis [8]. These burns may result in swelling of the oropharynx and laryngopharynx progressing ultimately to airway obstruction. When concern for inhalational burns is present, early intubation is important to contend with continued airway swelling that may obscure important landmarks making successful ETT placement very difficult (Figure 4).

Post-simulation evaluation

Following scenario participation, the learners completed an evaluation. Results are summarized in Table 7.

**Table 7:** Simulation participant evaluation results. Median response from Likert scale:  1 = strongly disagree; 2 = disagree; 3 = neutral; 4 = agree; 5 = strongly agree.

Question	Median (n)	Range
The simulation was realistic.	4.5 (15)	4-5
The simulation was clinically relevant.	5 (15)	4-5
The simulation helped me improve my overall comfort level in caring for critically ill patients.	4.5 (15)	3-5
The debrief provided valuable learning.	5 (15)	5
The debrief session was a safe and supportive environment.	5 (15)	5

## Discussion

A child presenting after involvement in a meth lab explosion is a rare event. Additionally, dealing with decontamination from a toxic chemical exposure and management of an inhalational injury in a pediatric patient are also rare events. These uncommon events may not be encountered clinically by all participants during training, so this simulated session gives learners crucial experience with a high-stakes medical condition. The goal in designing this scenario was to provide the learners with a realistic and challenging case in which they recognize and manage both a toxic chemical exposure and an inhalational injury. Additionally, we sought to provide the learners with an opportunity to practice and refine their leadership and communication skills.

The main challenge with the implementation of this case was that despite the development of symptoms in the EMS provider, participants had difficulty recognizing the presence of toxic chemical exposure. We decided not to apply any chemicals to the sim mannequin clothing to provide olfactory cues for toxic chemical ingestion due to concerns about its effects on the participants. Thus, recognition of a toxic chemical exposure is primarily dependent on cues from the actors in the case. As a result of this observation, the case was modified to include the possibility of the nurse also developing symptoms if the learners do not recognize cues from the EMS provider. Additionally, although the participants were familiar with the use of PPE and the need to bag contaminated clothing, most participants were unfamiliar with the logistics of water-based decontamination. Because of this directly following the simulation participants were taken on a tour of the hospital's water-based decontamination area and the process was reviewed. This additional teaching was very well-received by all participants and will be included in future simulations.

Future potential revisions of this simulation will be designed to directly address concerns raised by participants. Specifically, an additional faculty role of the observer was not originally included in the case but was requested by participants. This potential role was added to the case. The specific goals of the faculty observer would be to provide an objective assessment of the events of the case using the critical actions list (and to use this to help facilitate the debrief. Additionally, participants frequently commented on the importance of having hands-on practice for decontamination procedures so a visit to the ED decontamination bay was incorporated after the subsequent simulations. An additional future avenue for this case will be to increase the emphasis on difficult airway management. Potential avenues for this include using a simulator that can model airway edema and adding procedural skills for difficult airway management.

## Conclusions

Although this is not a frequent clinical presentation, this is one that can have very serious consequences if not properly managed, thus necessitating a venue in which providers can learn and practice their management skills. Simulation is a very effective way in which to provide this type of training. Overall this simulation was well received by the participants who felt it was clinically relevant and effective at improving their comfort level with caring for critically ill patients. The scenario in particular allows the learners to practice rapid assessments of critically ill patients, perform decontamination procedures, consider differential diagnoses, and perform advanced airway management skills. Experience in and knowledge of these interventions and considerations are key skills for all providers caring for children in a pediatric emergency department.
